# The Ethics of ‘Deathbots’

**DOI:** 10.1007/s11948-022-00417-x

**Published:** 2022-11-22

**Authors:** Nora Freya Lindemann

**Affiliations:** Institute of Cognitive Science, University of Osnabrück, Osnabrück, Germany

**Keywords:** Deathbots, Grief, Death, Ethics of AI, Chatbots

## Abstract

Recent developments in AI programming allow for new applications: individualized chatbots which mimic the speaking and writing behaviour of one specific living or dead person. ‘Deathbots’, chatbots of the dead, have already been implemented and are currently under development by the first start-up companies. Thus, it is an urgent issue to consider the ethical implications of deathbots. While previous ethical theories of deathbots have always been based on considerations of the dignity of the deceased, I propose to shift the focus on the dignity and autonomy of the bereaved users of deathbots. Drawing on theories of internet-scaffolded affectivity and on theories of grief, I argue that deathbots may have a negative impact on the grief process of bereaved users and therefore have the potential to limit the emotional and psychological wellbeing of their users. Deathbot users are likely to become dependent on their bots which may make them susceptible to surreptitious advertising by deathbot providing companies and may limit their autonomy. At the same time, deathbots may prove to be helpful for people who suffer from prolonged, severe grief processes. I caution against the unrestricted usage of deathbots and suggest that they should be classified as medical devices. This classification would not the least mean that their non-harm, as well as their helpfulness for people suffering from prolonged grief needs to be proven and that their potential for autonomy infringements is reduced.

## Introduction

Over the last years, Artificial Intelligence (AI) research and development has seen huge improvements, which allow for increasingly sophisticated applications. One field of AI research are chatbots, technological systems imitating human written or spoken natural language. It is possible to create individualized chatbots which mimic the writing and speaking behaviour of one specific person. This can be done by feeding the individual data of one person to an AI system, which is programmed to find patterns in the data to then imitate the writing behaviour of that person. The data which can be used for this ranges from emails to social media data, chat messages, video material and (digitalized) letters. In January 2021, Microsoft was granted a patent for such an individualized chatbot system. This chatbot could use the data of a person “to create or modify a special index in the theme of the specific person’s personality”, and hence could “respond like someone you knew” (Brown, 2021). One of the most likely applications of individualized chatbots is to create chatbots of the dead, ‘deathbots’ (Stokes, 2021). While Microsoft's general manager of AI, Tim O'Brien, assured that the technology will not be used (yet) due to ethical concerns and because of the “disturbing” character of this kind of application of the technology, the patent clearly shows the growing interest in the technological and financial potential of individualized chatbots – an opportunity which some start-up companies already try to seize (Duffy, 2021; Parker, 2014; Smith, 2021).

As the first deathbot developing companies enter the scene, it is pivotal to start thinking about the potential ethical implications of deathbots. Despite this, there is relatively little research on the ethics of deathbots (Stokes, 2021). The few existing ethical considerations of deathbots have two things in common: first they are for various reasons not fully convincing.^1^ Secondly, which is the main concern for this paper, their arguments are based on the human dignity of the deceased (Buben, 2015; Öhman & Floridi, 2017; Stokes, 2021). Instead of following this common point of departure, I propose to shift the focus of the ethical investigation of deathbots to the dignity and autonomy of bereaved users of deathbots. I do not mean to imply by this that the other considerations are irrelevant, but that they leave out the important focus of the ethical impact of deathbots on their users. While it is certainly important to consider the dignity of the deceased, it is even more urgent to analyse the ethical impact of deathbots on bereaved as they directly experience this impact in their life.^2^ This focus enables me to relate the ethical questions regarding deathbots more easily with questions of their influence on user autonomy and user dignity as they may have a strong impact on the grief processes of their users (Krueger & Osler, 2019, 2022). Finally, despite the shift in focus, the resulting view is compatible with some of the previously made claims about the dignity of the deceased mimicked by deathbots. Overall, this so far neglected perspective contributes a new layer to the existing debate.

The paper is structured as follows: in section two, I show that deathbots function as internet-enabled techno-social niches and have the potential to impact the grief processes of their bereaved users. Therefore, I argue in section three, they can have effects on the psychological well-being and dignity of their bereaved users. Moreover, they can decrease the autonomy of their users, as well as the dignity of the deceased. Thus, in section four, I propose that the usage of deathbots should be regulated. Specifically, I argue that deathbots could be considered to be medical devices for the treatment of people with a Prolonged Grief Disorder.

## (Online) Affect and the Grief-Shaping Capacities of Deathbots

### Deathbots as Internet-Enabled Techno-Social Niches

Deathbot interactions, just like online interactions in general, have the potential to shape the affective states of users (c.f. Krueger & Osler, 2019). Colombetti and Krueger (2015), among others, claim that affectivity is not just a passive process in which an individual undergoes bodily and experiential changes caused by external and internal factors, but that it also has an active component. Individuals can intentionally modify their own affective state. I can, for example, create different playlists to which I listen in different occasions: one for partying, one for relaxing after a stressful day and one I switch on while going jogging. Thus, I can manipulate my surroundings to create *affective niches* which are “instances of organism-environment couplings (mutual influences) that enable the realization of specific affective states” (Colombetti & Krueger, 2015, p. 1160). These affective niches can sometimes be enabled by the internet: they are *internet-enabled techno-social niches* (Krueger & Osler, 2019). Examples for this are, e.g., Instagram or fitness tracker apps. Internet-enabled techno-social niches have certain characteristic features which are due to their status as affective niches as well as to the specificities of the internet. These features include user trust, reliance that the scaffolding affects the user in the intended way, and entrenchment (Krueger & Osler, 2019). Only because the niches possess these properties, they have their distinctive regulative power over their users affects.

Deathbots represent internet-enabled affective niches according to Krueger and Osler (2019, p. 223), who note that the “Internet, by providing dynamic, ongoing interactions with chatbots offers a novel form of engineering the affective contours of our grief processes”. Therefore, deathbots can influence their user’s affect. While the creation of affective niches is often an intentional process, internet-enabled techno-social niches sometimes impact their users in unintended ways and can lead to overreliance and overregulation. The term overregulation, in this context, “is meant to pick out how the Internet, by allowing us constant access to highly tailored and individualized scaffolding and niches, make us reliant not just on specific forms of emotion regulation but upon emotion regulation itself” (Krueger & Osler, 2019, p. 227). As deathbots allow to seemingly continue chatting with a deceased person, it is likely that they will be used by people who urgently wish they could keep talking with a deceased person: grieving bereaved who lost a dear friend, partner, or family member.^3^ It is no surprise that deathbots have been called “griefbots”, implying that they may take part in grieving or may shape grief processes (Bassett, 2018b).^4^ Deathbots can therefore impact the grief of their users in the intended way (e.g. helping to deal with grief)—but may also make their users reliant on their bots to regulate their grief. Users may start to feel that they *need* their deathbot to deal with the grief they are experiencing. The bot, then, functions not only to regulate their affect but also to *dys*regulate it, meaning that it can lead to users over-relying on the bot for the regulation of their affects such that they constantly need it for emotion regulation (especially for regulating grief). This may lead to a situation in which users feel unable to regulate their affect without the bot (cf. Krueger & Osler, 2019). Moreover, the term dysregulation is meant to signify that our affects can be regulated in the environment (and thus also by a deathbot) in unintentional ways which can lead to a state of affective vulnerability where users may be open to manipulation and can experience affective harm (cf. Krueger & Osler, 2019). This dysregulation of affect may have stark consequences on the affective and psychological well-being of bereaved users, as I will argue in more detail in Sect. ‘The Ethics of Deathbots’.

### Deathbots Acting as Continuing Pseudo-Bonds

In addition to the potential of deathbots to dysregulate their user’s affect, deathbots may act as continuing pseudo-bonds between bereaved and deceased. Psychological theories hold that in grieving the bereaved need to re-structure their relationship and bond with the deceased as the end of the physical relationship with the dead person needs to be accepted (Klass & Steffen, 2017). However, a continued—though transformed—emotional bond to the deceased is mostly kept after the death of a person (Rothaupt & Becker, 2007). Deathbots can impact these continuing bonds, as this example by Krueger and Osler (2019) illustrates: Stella, a bereaved granddaughter, uses a deathbot to continue to talk with her late grandmother Jean. Stella often talks to the Jean deathbots about her life as a nightly ritual that she finds comforting. Krueger and Osler describe the conversation of Stella with the deathbot of her grandmother in the following way: “She uses this time [of her nightly deathbot interaction] to talk about her day, share secrets, cultivate a sense of security at the sound of Jean’s soothing voice, and feel as though she’s preserved a continuing connection with her dead grandmother” (2019, p. 215). Because she continuously interacts with the bot, she becomes entrenched in the affective niche created by these interactions. She establishes the bot as a connection with her late grandmother and starts to trust the deathbot to *be* the emotional foundation of her continuing connection—her continuous bond—with her grandmother. I will refer to the continuing bonds between bereaved and deceased which are mediated in this way as continuing pseudo-bonds.

While Krueger and Osler consider the potential of deathbots to sustain the bond between bereaved and deceased as a positive development, a continuing bond mediated by a deathbot has different qualities than a continuing bond which is not mediated by, and dependent on, a deathbot. There are decisive qualitative differences between, for example, reading old letters and mails of a deceased versus using a deathbot of her. Deathbots allow for an *interaction* in a way that mere left letters do not allow for. Only deathbots can answer to specific, new questions of the bereaved and create novel messages which would not be available without the bot as they cannot be found in old letters. Moreover, in the usual grief process the formerly *external* (i.e. between two living people) bond is *internalized* (Fuchs, 2018). A non-mediated continuing bond with the dead person is a feeling, a comforting, inner presence of the dead (Fuchs, 2018). When using a deathbot, the bond may stay partly externalized as it is (partly) formed between the bereaved and the deathbot. An externalized bond, however, is less secure than an internal bond. Having an internally attributed presence of the deceased person gives the security of not losing the bond with them. The attachment is less secure if the bond is technologically externalized. If Stella experiences the deathbot as a reliable and trustworthy continuing connection to her late grandmother, she *relies* on it to sustain her continuing bond with her. This reliance makes her *dependent* on the bot, as losing the bot would mean losing a crucial part of the connection to her grandmother. This strong dependence on the bot, as I will argue in more detail in Sect. ‘Deathbots and Autonomy’, can reduce her autonomy to act according to her own needs and wishes and makes her susceptible to commercial exploitation by deathbot providers.

## The Ethics of Deathbots

### Deathbots and Dignity

#### Grief and Well-Being

Dignity, autonomy, and well-being are some of the most pressing ethical issues concerning the impact of deathbots on bereaved users and will be discussed in detail in this paper. However, other ethical issues, such as privacy, accountability, and data protection, may also be important and could be addressed in future research. Due to their affect-shaping capacities and their impact on grief processes, deathbots can violate the affective and psychological well-being of their bereaved users. Grief is a multi-facetted emotion which comprises many singular emotions like sadness, a feeling of longing for the other person, loneliness, guilt, or anger that the other person has died. These different emotions hang together to form a pattern in grief (Goldie, 2011). Grief is necessarily temporally extended—if an emotion would only last for a few seconds, it would not be grief (Wittgenstein, 1968). Therefore, grief can be understood as a multi-faceted, complex, heterogenous, changeable and transforming process (Fuchs, 2018; Ratcliffe, 2016, 2017).^5^ In this process, the bereaved experiences a feeling of ambiguity and uncertainty between a “presentifying and a ‘de-presentifying’ […], presence and absence, between the present and the past, indeed between two worlds they live in—an ambiguity which may also manifest itself in being painfully torn between acknowledgment and denial of the loss” (Fuchs, 2018, p. 44). The ‘two worlds’ constitute the pre- and the post- death world experience by the bereaved. In successful grieving, the two experienced worlds merge. The process of merging entails the transformation of the continuing bond between bereaved and deceased from an external to an internal presence of the dead person. Moreover, in a successful grief process the death of the deceased person is fully (not only intellectually) acknowledged (Fuchs, 2018). The deceased needs to move from an imagined status of not-being-alive-but-also-not-being-dead to an accepted status of being dead. Grief thus constitutes a recognition of loss.

While there is no empirical evidence on the impact of deathbots on grief processes, it seems likely that they may foster the ambiguous in-between two worlds status bereaved experience. This is because deathbots, through their imitation of the deceased person’s way interaction and communication style, make the deceased appear not quite gone. A pretending that the deceased person is not really gone even though the bereaved has the intellectual knowledge of the person’s death can happen in grief in general (c.f. Fuchs, 2018) and may be unnaturally temporally extended by deathbots. Writing with the bot, which texts just like the deceased person did, makes pretending that the deceased person is not quite gone easy. For example, I may turn to the deathbot mimicking my deceased father to write to it in situations in which I would have turned to message my father previously to his death. While I have the intellectual knowledge that he is dead, receiving messages from the bot that sound like coming from him makes it easy for me to pretend that he is still reading my messages. Whenever I strongly feel grief, I turn to the deathbot to ease my pain. In this situation, I do not need to fully adapt to a world without my father. Thus, I do not fully acknowledge the death of my father and do not fully adapt to the world without him. Deathbots, therefore, have the potential to prevent the two experienced worlds to merge and thus the bereaved from re-orienting in the post-death world. Through this, deathbots might (at least temporarily) hinder an otherwise successful grief process.

If this merging of the worlds, the process of accepting the death and transforming the bond with the deceased, does not take place, the bereaved may experience an unsuccessful grief process. “Successful” in this context means that the bereaved can live a fulfilled life again at some point after their loss. Unsuccessful grieving may inhibit this possibility for a prolonged period of time, sometimes for the rest of the bereaved person’s life. Some bereaved who experience unsuccessful grieving develop a Prolonged Grief Disorder (PGD), a recognized psychological disease which leads to a reduced quality of life and mental health problems (Boelen & Prigerson, 2007). Bereaved struggling with PGD experience “intense, prolonged and complicated grief, characterized by extreme separation distress, preoccupation with the loss, and inability to function in major life roles across a period of many months or years” (Neimeyer & Thompson, 2014, p. 4). PGD is a disease which occurs without the use of deathbots. However, while there is no empirical evidence to the prevalence of PGD in deathbot users, through their potentially negative impact on the grief process of bereaved who may otherwise have had a successful grief process, they may pose a risk to the psychological and emotional health of their users.^6^ This marks an intrusion on the dignity of the bereaved, as human dignity requires that a human’s psychological health is not intentionally harmed and that measures are taken to prevent unnecessary psychological harm. I understand dignity as a subjective experience here which is realized through the individual’s experience of it (Mattson & Clark, 2011). Psychological integrity is therefore essential for the dignity of the bereaved. Deathbots thus pose a risk on the dignity of their users and require a normative framework to guide an ethical usage of them.

#### Unilateral Emotional Bonding

Deathbots may lead to unilateral emotional bonding with an AI system and impact the relationship users have with other humans. If users create an internet-enabled techno-social niche with their deathbot, they become highly entrenched in the niche and rely on the bot to scaffold their affect. At the same time, humans easily anthropomorphise technological devices and—unintentionally and unconsciously—ascribe human characteristics and traits to them (Bartneck et al., 2021; Duffy, 2003; Kim & Sundar, 2012). Deathbots, which exhibit very human characteristics as they imitate a human’s writing behaviour, can be easily anthropomorphized. Users can therefore quickly develop an emotional attachment to their bots, which is, however, is always unidirectional. While users may feel like their deathbots emotionally answer them (through their carefully programmed outputs), deathbots naturally cannot emote and do not develop an emotional bond towards their users as they lack the capacity to do so. The emotional bond is therefore necessarily shallower than between two people. The impact this has on users, and on user’s relationship to other humans, should be the object of further research. It seems plausible that it may impact on both.

At the same time, deathbots are always available, always answer, are always patient and mostly answer in an expected and desired way. Users may become accustomed to such a behaviour and expect it in their interactions. This expected behaviour may be transferred to human–human interactions and will necessarily be disappointed. In some cases, the idealised interactions with the bot may become so much of a benchmark for users that they may start to question human–human interactions as they do not reliably answer in the expected way (c.f. Bartneck et al., 2021 for a discussion of unilateral emotional bonding with AI). Thus, deathbots have the potential to negatively impact human–human interactions. This may lead users to become even more dependent on the bot for their emotion-regulation. Moreover, it may bear the danger that some deathbot users become socially isolated, leading to a further disturbance of the emotional and psychological integrity of their users.

#### The Inherent Value of Digital Remains

As mentioned before, previous ethical theories of deathbots start with the assumption that deathbots infringe the dignity of the deceased. For example, Öhman and Floridi (2017) claim that the informational body of a person—all of their informational and digital traces—is their identity, as it holds all (or at least most) of their relevant personal information. The personal identity of a person defines that person and therefore essentially belongs to that person. Having the control over one’s own personal identity is an essential human condition and an essential human right. If the control over one’s personal identity is taken away from someone, therefore, an essential aspect of what it means to be human is taken away from the person. An intentional changing of the informational body means that one is no longer the “master of one’s existence, of one’s own ‘journey’ through the world” (Öhman & Floridi, 2017, p. 650). The informational body of the deceased, Öhman and Floridi claim, is likely to be changed if it is turned into a deathbot. Deathbots providers are commercial companies and therefore have an interest in creating as much revenue as possible. Revenue will most likely be created either through a continuous monthly subscription fee, through using the data of deathbot users for targeted advertisement, and/or through selling their user’s data to third party companies.^7^ Thus, Öhman and Floridi (2017, 2018) argue, it is likely that companies intentionally adapt the digital remains of the deceased to make their deathbot impression more consumable. For example, a formerly introverted person whose digital remains are fed into a deathbot may be depicted as more outgoing, thus enticing the deathbot user to interact with it longer. This change of the impression of the deceased leads to the deceased being remembered in an altered way by the living. Öhman and Floridi argue that through this an essential part of being human, the possibility to shape one’s own personal identity, is taken away from the deceased in deathbots. This, they claim, infringes the dignity of the deceased and is therefore an ethical wrong. As already mentioned in the introduction, arguments against it have been laid out elsewhere (Lindemann, 2022). However, the regulatory framework for the use of deathbots Öhman and Floridi propose is fruitful.

The authors argue that the regulatory framework for archeological exhibitions of deceased should also guide a framework for the treatment of digital remains. In both cases, the ownership of the remains may be difficult to determine, and the remains are displayed or used for the consumption by the living. The exhibition regulation stipulates that human remains must be treated such that the dignity of the remains is ensured. The human dignity “requires that digital remains, seen as the informational corpse of the deceased, may not be used *solely* as a means to an end, such as profit, but regarded instead as an entity holding an *inherent* value” (Öhman & Floridi, 2018, p. 319 original emphasis). I will come back to this in Sect. ‘An Ethical Framework for Deathbots’ in which I put forward an ethical framework for the usage of deathbots. While I propose to analyze the ethical aspects of deathbots from the perspective of their bereaved users, the framework for the usage of deathbots I suggest is compatible with Öhman and Floridi’s theory which is based on considerations of the dignity of deceased.

### Deathbots and Autonomy

#### Emotion-Regulation

In addition to the likely negative impact of deathbots on the dignity of bereaved and deceased, deathbots may also violate the autonomy of the bereaved. This is largely due to the impact of deathbots on grief processes. If users rely on their deathbots to scaffold their grief and to sustain a continuing bond to the deceased, they develop an emotional reliance on their deathbots. While people who use internet-enabled techno-social niches generally rely on them to regulate their affects as intended, the specific features of grief make a sudden stop of deathbots usage extremely distressing for the bereaved. They may feel like they are thrown back to earlier stages of their grief process.^8^ As grief is a painful and challenging process, users likely start to feel the necessity to keep using the deathbot. Deleting or quitting the usage of the deathbot may then not be a viable option anymore for some users, which reduces the users’ ability to act independently according to their own needs and wishes. This leads to a diminishing of the user’s autonomy.

### Externalized Pseudo-Bonds

The user autonomy may be further diminished because deathbots can act as externalised pseudo-bonds between bereaved and deceased. If they do so, deathbot users need their deathbots in order to feel in touch with their deceased relative or friend. They rely on it to feel the continued presence of the deceased. The deathbot, therefore, decisively shapes how they can deal with the grief and changes the re-negotiation of the bond between deceased and bereaved. Bereaved users cannot stop using the bot without losing, or at least fearing to lose, the continued bond with the dead. While this feeds into the felt inability of deathbot users to delete their bot, it additionally makes bereaved users vulnerable to sale strategies of deathbot providers. Deathbot users are then, for example, unlikely to stop using the deathbot if the fee for it raised - which deathbot companies may exploit.

Moreover, once users are highly entrenched in their deathbot, the deathbot is likely to be perceived as trustworthy. Users may not be aware then that the deathbot is provided by a commercial company and that their interaction with the bot is likely to be saved by the provider. Through continued interaction with the bot, users reveal a lot of personal information about themselves which companies can (mis)use to make profit. For example, deathbot providers could use that information to infer in which type of conversation the user interacts longest, to then employ that knowledge to keep the user interaction with the bot longer than it would have otherwise been or, may program the deathbot to convince their users to buy something they otherwise would not have bought. For example, a deathbot may have collected a lot of information about its user, which can be used to analyse the user’s favourite T-shirt style, brand, and colour. The deathbot may then propose its user to buy a certain T-shirt which fits these categories. Imagine that Stella from the previous example is shown a link to a T-shirt by her Jean deathbot with the caption “I think this would look gorgeous on you”. She is likely to at least have a look at the product, as she may trust the taste of her deceased grandmother who she senses in the bot’s messages. As a more extreme example, one could imagine the deathbot recommending (and influencing) the voting behaviour of its user.

While this can of course happen through individualized advertisement too, making the deathbot advertise certain products or ways of behaviour may be highly persuasive for the user. The deathbot then acts as a persuasive AI, meaning that it can influence the (shopping) behaviour of its user (cf. Bartneck et al., 2021). Thus, users may do or buy something they otherwise would not have bought or done. This may diminish the ability of bereaved to act autonomously and uninfluenced according to their own needs and wishes.

## An Ethical Framework for Deathbots

I have so far concentrated my discussion on people who would experience successful grieving without the usage of deathbots. Bereaved who developed a prolonged grief disorder (PGD), however, did not undergo a process of successful grieving, re-orientation and re-negotiation of the continuing bonds without using deathbots. Despite the likely negative consequences of the usage of deathbots which I laid out above, there are also hints that deathbot usage may help people in this state of severe and prolonged grief. In modern psychological grief therapy, there are two different narratives of the bereaved which are approached: the first is the story of the event of the death itself (e.g. the circumstances in which the person died) which needs to be processed. The second is the story of the relationship between deceased and bereaved and entails an effort to access and reorganize the continuing bond between bereaved and deceased (Neimeyer & Hooghe, 2018). Virtual encounters may help with reorganizing the continuing bond as this example shows: a bereaved Korean mother with prolonged grief was able to interact with an avatar of her deceased daughter in a pre-scripted VR environment. She seemed to have found it distressing but also comforting and reported feeling that she could finally say goodbye to her daughter through this one time virtual meeting (Simon, 2015; The Korea Times, 2020). This helped her in her unfinished, year-long grief process. This encounter has quite different features than the usage of deathbots as this was a single interaction with an avatar, the avatar could not behave or answer in different ways than it was previously programmed to and the interaction took place in a VR environment. Nevertheless, it shows that technological devices can potentially also have a positive impact on bereaved people struggling with prolonged grief.

Thus, deathbots may prove to be a way for bereaved to deal with their grief while they may at the same time pose serious ethical issues on the dignity, autonomy and psychological well-being of bereaved. As I argued above, they are likely to impact negatively on the bereaved without any regulations posed on their usage and on the companies selling them. Therefore, I propose that the implementation, provision, and usage of deathbots should be regulated. This regulation should ensure that the dignity and autonomy of the bereaved is upheld and that the dignity of the deceased is maintained. At the same time, the potential of deathbots to support bereaved people suffering from PGD should be taken into account.

Deathbots, I therefore recommend, should be further discussed as a topic of a regulative legal framework as they may pose several (ethical) risks if they are available without restriction. In particular, the potential to classify deathbots as medical devices could be further explored. While not banning deathbots entirely, this would take into account the indicated possible positive outcomes of deathbot usage in the treatment of PGD, while also acknowledging their inherent ethical dangers. The classification of deathbots as medical devices for the treatment of PGD would have several concrete consequences. Deathbots would then need to go through several phases of testing before they could be widely used (BMBF, n.d.). They would have to prove their non-harm to all users as well as their benefits in treating PGD before they may be used. In addition, deathbots would not be available for people who are not diagnosed with PGD, which includes people who are newly bereaved and just start the process of re-orientation in a changed world.

Classifying deathbots as medical devices could lead to the avoidance of the most pressing ethical issues regarding the usage of deathbots which were outlined above. To start with, deathbots can diminish the autonomy of bereaved users as they can become dependent on the bot for their emotion regulation. Moreover, deathbots may act as continued pseudo-bonds which further inhibits the autonomy of users to delete their bot. If deathbots are understood as medical devices, they would only be allowed for the usage under psychological or psychiatric supervision. In this usage, then, patients as well as medical staff should be aware of the potential of deathbots to limit the autonomy of users and should ensure that it is kept as low as possible. Measures need to be taken to ensure that bereaved may not become (overly) dependent on their deathbots. For example, through a limited and non-constant use of deathbots, they could be used as a way of re-negotiating the continuing bonds with the deceased without constructing the deathbots as the only means to sustain a continuing bond.

Another aspect that could lead to an infringement of user autonomy is the influence of deathbots on users’ consumption behaviour which is tied to the commercial nature of deathbot developing companies. Even if deathbots are understood as medical devices, the providing companies themselves would (most likely) still be commercial endeavours and the implementation and usage of a deathbot would still cost the user (and/or their health insurance) money. The ways in which a deathbot provider would be able to make money with the bot, however, would be limited. For example, surreptitious advertising, as in the example of the deathbot advertising a certain T-shirt, would not be possible as the users data would then be classified as patient’s data, which is protected by higher data protection regulations than regular user data (European Patients Forum, n.d.). In addition, it could be prohibited to change the depiction of the deceased through the bot to make the bot becomes more addictive. Thus, categorizing deathbots as medical devices could be a valuable step to avoid a diminishing of user autonomy.

Regardless of the issue of user autonomy, I drew on Öhman and Floridi (2017, 2018) who propose that digital remains should be treated like archaeological remains and that, therefore, they should be seen as having an inherent value and not be treated solely as a source of consumption for the living. If digital remains are used to generate a deathbot, they are inevitably consumed by the living. If deathbots would be understood as a medical device, they would have the instrumental value of helping bereaved in their struggle with PGD and would thus not *solely* be a source of consumption. Moreover, this framing automatically excludes certain ways in which deathbots could theoretically be implemented. For example, it avoids scenarios in which the digital remains of celebrities are posthumously turned into deathbots, which are then available to be bought and used by everyone. In this case, the digital remains would only be a source of consumption by the living. If a deathbot is classified as a medical device, its use would be limited to people who have had a valued relationship with the person it depicts. The digital remains at its basis are thus of valued interest for the bereft user and the deathbot is seen as having the potential value of helping the bereaved in their grief process. The inherent value of the digital remains is upheld and their complete capitalization would be restricted.

## Conclusion

Up to today, no legal framework for the use of deathbots has been issued anywhere (c.f. Stokes, 2021). Everyone who can implement a deathbot is allowed to program, use and sell deathbots. While deathbots are not commonly used yet, they yield the potential to be widely used in the near future without a regulative framework. This points to the pressing issue to think about the ethics of deathbots *now* to proactively shape their future use. In this paper, I propose to shift the focus of the ethical analysis of deathbots from considerations of the dignity of the deceased to the dignity and autonomy of bereft users of deathbots. Deathbots function as internet-enabled techno-social niches and can therefore have a strong impact on the affective life, especially the grief process, of their users. Due to the specific characteristics of grief, deathbots may lead to an infringement of the affective and psychological well-being of their bereaved users. Moreover, they may violate the dignity and autonomy of their users. To avoid this, I propose that we need to start discussing regulations on the usage of deathbots. To start this discussion, I suggest that deathbots could, for example, be classified as medical devices for the potential treatment of PGD, which would not the least mean that their non-harm to their users would need to be tested before they may be used. Understanding deathbots as medical devices means that infringements on the dignity and autonomy of deathbot users, which could otherwise occur, are prevented. At the same time, the digital remains of the deceased would be seen as containing an inherent value and are not expropriated completely from their original producers, thus avoiding a violation of the dignity of the deceased which have been laid out by Öhman and Floridi. Thereby, several potential ethical concerns could be accounted for.

## Footnotes

1 While a thorough discussion of this is out of the scope of this paper, this issue has been discussed in detail in Lindemann (2022).

2 Krueger and Osler (2022) argue from within the philosophy of emotions that deathbots may impact grief processes of their users and seem to have a rather positive outlook on deathbots, however, they do not discuss this ethically.

3 The first functioning deathbot was designed for this reason. When the tech entrepreneur Roman Mazurenko suddenly died, his friend Eugenia Kuyda (working in AI development) decided to create a deathbot from Romans extensive bulk of text messages to deal with her grief (Bloomberg Nagels, 2016; Quicktake, 2020).

4 In this paper the term ‘griefbot’ will not be used as it seems to imply that those bots necessarily are, or should be, used for grieving. Instead, the term ‘deathbot’ is used as a more encompassing and neutral term and as an abbreviation of the expression ‘chatbots of the dead’.

5 Understandings of grief and grief practices are culturally dependent (Sofka et al., 2012) and therefore it is important to note that this paper and the sources herein cited on grief come from a European/Anglophone perspective.

6 This is connected to the issue of safety as I will discuss further in “An ethical framework for deathbots” section.

7 This is for example the business model of Facebook which earns money with targeted advertisement. The more time a user spends on the platform, the more advertisement can be displayed and the more money Facebook earns (Reichert, 2012). the main option after a user’s death on Facebook is to turn their profiles into a ‘memorial page’ rather than deleting it which makes it more likely for bereaved users to spend time on the platform while they interact with the profile of the deceased. An ethical discussion of this is out of the scope of this paper but should be addressed in future research.

8 If a formerly reliable deathbot suddenly stops working (properly), this experience may cause the feeling of a ‘second loss’ in users. The concept of a ‘second loss’ was introduced by Bassett (2018a) and refers to the fear of losing the (digital) data of a deceased person by the bereaved. In a qualitative study, she found that many bereaved had a fear of a ‘second loss’ which would throw them back to earlier stages of their grieving process. A second loss can of course also happen in the non-internet realm. However, digital remains often have a much larger quantity and are less tangible.
